# Social media content analysis on twitter to explore public perceptions regarding pathological social withdrawal (hikikomori)

**DOI:** 10.1192/j.eurpsy.2021.181

**Published:** 2021-08-13

**Authors:** V. Pereira-Sanchez, M. Alvarez-Mon

**Affiliations:** 1 Psychiatry And Medical Psychology, Clinica Universidad de Navarra, Pamplona, Spain; 2 Child And Adolescent Psychiatry, NYU Grossman School of Medicine, New York, United States of America; 3 Department Of Psychiatry, University Hospital Infanta Leonor, Madrid, Spain

**Keywords:** social withdrawal, social media, Hikikomori, twitter

## Abstract

Hikikomori is a form of severe social withdrawal, initially described in Japan and recently reported in other countries around the world. Individuals with hikikomori shut themselves in their homes with minimal interaction with society and little participation in school or the workforce. The nature of hikikomori makes the individuals suffering it a hard-to-reach population. While hikikomori was described in Japan much before the ‘digital revolution’ of the 2000s, the internet, social media, and online gaming have radically changed the way people interact. This may be particularly true among hikikomori who spend much time online for entertainment or social interaction. Given this, the online world has been proposed as an accessible gateway to reach and support individuals with hikikomori. This talk will present and discuss the results of the Twitter-hikikomori international studies, conducted between 2018-2020 and led by Dr. Pereira-Sanchez, which employed social media mixed quantitative-method analyses to characterize the public conversations related to hikikomori on the social media platform Twitter in several Western languages and Japanese. As for the results, Twitter data provided evidence that hikikomori extends well beyond Japan examining, and showed that tweets in Japanese are more often are related to personal anecdotes, whereas tweets in Western languages are more often related to hikikomori as a medical issue. Apart from the results of the content analyses studies have been a proof of concept on the use of social media contents to investigate a phenomenon affecting a hard-to-reach population, which may inspire future online-based efforts to better support these populations.

**Disclosure:**

No significant relationships.

